# Metformin prevents methylglyoxal-induced apoptosis by suppressing oxidative stress in vitro and in vivo

**DOI:** 10.1038/s41419-021-04478-x

**Published:** 2022-01-10

**Authors:** Gang Wang, Yanan Wang, Qinzhi Yang, Chunrong Xu, Youkun Zheng, Liqun Wang, Jianbo Wu, Min Zeng, Mao Luo

**Affiliations:** 1 Collaborative Innovation Center for Prevention and Treatment of Cardiovascular Disease of Sichuan Province, Drug Discovery Research Center, Southwest Medical University, Luzhou, China; 2 Laboratory for Cardiovascular Pharmacology, Department of Pharmacology, School of Pharmacy, Southwest Medical University, Luzhou, Sichuan China; 3 School of Pharmacy, Chongqing Medical University, Chongqing, China; 4 Department of Pharmacy, The Affiliated Hospital of Southwest Medical University, Luzhou, Sichuan China

**Keywords:** Apoptosis, Stress signalling

## Abstract

Methylglyoxal (MGO) is an active metabolite of glucose and plays a prominent role in the pathogenesis of diabetic vascular complications, including endothelial cell apoptosis induced by oxidative stress. Metformin (MET), a widely prescribed antidiabetic agent, appears to reduce excessive reactive oxygen species (ROS) generation and limit cell apoptosis. However, the molecular mechanisms underlying this process are still not fully elucidated. We reported here that MET prevents MGO-induced apoptosis by suppressing oxidative stress in vitro and in vivo. Protein expression and protein phosphorylation were investigated using western blotting, ELISA, and immunohistochemical staining, respectively. Cell viability and apoptosis were assessed by the MTT assay, TUNEL staining, and Annexin V-FITC and propidium iodide double staining. ROS generation and mitochondrial membrane potential (MMP) were measured with fluorescent probes. Our results revealed that MET prevented MGO-induced HUVEC apoptosis, inhibited apoptosis-associated biochemical changes such as loss of MMP, the elevation of the Bax/Bcl-2 ratio, and activation of cleaved caspase-3, and attenuated MGO-induced mitochondrial morphological alterations in a dose-dependent manner. MET pretreatment also significantly suppressed MGO-stimulated ROS production, increased signaling through the ROS-mediated PI3K/Akt and Nrf2/HO-1 pathways, and markedly elevated the levels of its downstream antioxidants. Finally, similar results were obtained in vivo, and we demonstrated that MET prevented MGO-induced oxidative damage, apoptosis, and inflammation. As expected, MET reversed MGO-induced downregulation of Nrf2 and p-Akt. In addition, a PI3K inhibitor (LY-294002) and a Nrf2 inhibitor (ML385) observably attenuated the protective effects of MET on MGO-induced apoptosis and ROS generation by inhibiting the Nrf2/HO-1 pathways, while a ROS scavenger (NAC) and a permeability transition pores inhibitor (CsA) completely reversed these effects. Collectively, these findings broaden our understanding of the mechanism by which MET regulates apoptosis induced by MGO under oxidative stress conditions, with important implications regarding the potential application of MET for the treatment of diabetic vascular complications.

## Introduction

Diabetes mellitus (DM) is a type of metabolic disease characterized by hyperglycemia, which leads to a high risk of developing cardiovascular diseases [1, 2]. Evidence suggests that vasculopathy is one of the main causes of high mortality and disability associated with DM [3–5]. Endothelial cells (ECs) that form the inner lining of all blood vessels can safeguard transport logistics, regulate vascular tone, and control vascular permeability, which are the central components of the body’s immune and vascular systems [6, 7]. Intravascular homeostasis disorder caused by EC injury, overactivation, and dysfunction has been considered to be the major initiating cause in the pathogenesis of vascular complications in DM [8–10]. Several studies have suggested that ECs may be subject to oxidative stress that can perturb cellular components involved in intracellular signaling transduction pathways, resulting in cell proliferation or apoptosis [11–14].

Methylglyoxal (MGO) is a highly reactive dicarbonyl metabolite produced by glycolysis and has been found to serve as an advanced glycation-end product (AGE) precursor formed by the fragmentation of triose phosphates under hyperglycemic conditions, with subsequent activation of the receptor of AGEs in ECs [15–17]. Several reports have shown that plasma MGO levels in diabetic patients accumulate abnormally in multiple tissues and organs and play a prominent role in the pathogenesis of diabetic vascular complications [18–20]. MGO can induce cell damage, cytotoxicity, inflammation, and apoptosis, mainly through reactive oxygen species (ROS) generation, but the specific mechanism remains unclear. Metformin (MET) is an oral antidiabetic biguanide agent derived from *Galega officinalis* that not only reduces blood glucose levels and improves insulin sensitivity but also has benefits against cardiovascular diseases [21–24]. Several studies have shown that MET appears to reduce excessive ROS production and limit early apoptosis [25, 26]. Whether MET can reduce MGO-induced EC apoptosis, what roles MET and MGO play, and how MET and MGO interact with each other in the context of the cardiovascular protective effects of MET remain to be determined. Accordingly, targeted inhibition of related signaling pathways, particularly ROS signaling, may be effective in the treatment of diabetic cardiovascular diseases.

In the present study, we elucidated the protective effect of MET on MGO-induced apoptosis of human umbilical vein endothelial cells (HUVECs) in vitro, as well as the inhibition effect of MET on MGO-induced vascular injury and apoptosis in vivo. We further explored the molecular mechanism, that is, the reduction of oxidative stress and apoptosis mediated by the PI3K/Akt-mediated Nrf2/HO-1 signaling pathway.

## Materials and methods

### Chemicals, reagents, and antibodies

MGO and MET were purchased from Sigma-Aldrich Co. (St. Louis, MO, USA). Terminal deoxynucleotidyl transferase dUTP nick end labeling (TUNEL) kits, 4′, 6-diamidino-2-phenylindole (DAPI), enhanced bicinchoninic acid (BCA) protein assay kits, total superoxide dismutase (SOD) assay kits, catalase (CAT) assay kits, glutathione peroxidase (GSH-Px) assay kits, malondialdehyde (MDA) assay kits, 2’,7’-dichlorofluorescein diacetate (DCFH-DA), 3-(4,5-dimethyl-2-thiazolyl)-2,5-diphenyl-2H-tetrazolium bromide (MTT), N-acetyl cysteine (NAC), and LY-294002 (LY) were purchased from Beyotime Biotechnology (Shanghai, China). Methylglyoxal ELISA kits were obtained from Blue Gene Biotech (Shanghai, China). Annexin V-fluorescein isothiocyanate (FITC) and propidium iodide (PI) apoptosis detection kits and 5,5’, 6,6’-tetrachloro-1,1’, 3,3’-tetraethylbenzimidazolcarbocyanine iodide (JC-1) were purchased from BD Biosciences (San Diego, CA, USA). Antibodies against phospho-Akt, Akt, Bcl-2, Bax, cleaved caspase-3, and β-Actin were obtained from Cell Signaling Technology (Beverly, MA, USA). Antibodies against Nrf2 and HO-1 were purchased from Santa Cruz (CA, USA) and Proteintech Group (Wuhan, China). Cyclosporin A (CsA) was purchased from Gene Operation (Ann Arbor, Michigan, USA). Enhanced chemiluminescence (ECL) kits were purchased from Merck Millipore (Darmstadt, Germany). All other chemicals and reagents were purchased from Sigma unless otherwise stated.

### Cell culture

HUVECs were obtained from ScienCell (Carlsbad, CA, USA) and cultured in medium 200 (Gibco, Carlsbad, CA, USA) supplemented with low-serum growth supplement (Cascade Biologics, Portland, OR, USA). Cells were maintained at 37 °C in a humidified incubator containing 5% CO_2_. Cells at passages 3–7 were used for all experiments

### Cell viability

To determine the optimal drug concentration, cell viability was determined using MTT assays based on the manufacturer’s instructions. The cells were seeded in 96-well plates at a density of 2 × 10^4^ cells/well, incubated for 24 h, changed to a fresh medium containing various concentrations of MET (0, 0.1, 1, 10, 20, and 50 mM) for 2 h, and then stimulated with MGO (10, 50, 100, 200, and 500 μM) for 24 h. After that, the absorbance was measured at 570 nm with a Spectra Max M5 microplate reader (Molecular Devices, Sunnyvale, CA, USA). Data represent the mean ± SD of three independent experiments with five replicates in each experiment.

### Cell apoptosis assays

HUVECs were seeded in 6-well plates at a density of 5 × 10^5^ cells/mL and treated with MET (0, 0.1, 1, and 10 mM) for 2 h, followed by stimulation with MGO (200 μM) for 24 h. In some experiments, HUVECs were incubated with NAC (10 mM), CsA (1 μM), LY (50 μM), or vehicle control for 2 h before MGO (200 μM) treatment. Next, the cells were collected and washed with cold PBS, and the cells were resuspended in 1× binding buffer with Annexin V-FITC and PI at room temperature (RT, 25 °C) for 15 min according to the manufacturer’s instructions using an Annexin V-FITC Apoptosis Detection Kit. Then, the percentages of apoptotic cells were analyzed using a flow cytometer (Becton-Dickinson, CA, USA). Data were calculated with Cell Quest Software (Becton-Dickinson, CA, USA).

In addition, TUNEL staining was performed to detect the apoptosis index in vitro according to the manufacturer’s instructions using a commercially available kit. Briefly, HUVECs were fixed with 4% paraformaldehyde for 30 min and permeabilized with 0.1%Triton X-100 for 5 min. After washing with PBS twice, the cells were incubated with 50 μL TUNEL reaction fluid in a humid environment at 37 °C for 1 h. Finally, the results were observed using fluorescence microscopy (Olympus, Japan) and calculated using ImageJ software (NIH, Bethesda, USA). The number of TUNEL-positive nuclei per field was counted in five randomly micrographs for each sample. The numbers of TUNEL-positive HUVECs and total cells were counted, and apoptosis was evaluated by the ratio of positive cells to total HUVECs.

### Measurement of intracellular ROS levels

Intracellular ROS levels were measured using DCF-DA as a fluorogenic dye. Briefly, HUVECs seeded in 6-well plates were pretreated with MET (0, 0.1, 1, and 10 mM) for 2 h, followed by stimulation with MGO (200 μM) for 1 h. Cells were washed with medium without additives and subsequently loaded with approximately 500 μL of DCFH-DA (10 μM) at 37 °C for 30 min in the dark. After incubation, the cells were washed twice with 1× PBS to remove any excess DCF-DA that had not penetrated into the cells. Next, the fluorescence of the cells was measured using a Spectra Max M5 microplate reader (Molecular Devices, Sunnyvale, CA, USA) (485/530 nm), and fluorescence images were captured with an EVOS inverted microscope (AMG, Mill Creek, WA, USA).

### Measurement of intracellular SOD, CAT, GSH-Px, and MDA levels

HUVECs (1 × 10^5^ cells/well) were seeded in 6-well plates and grown to confluence. Then, the cells were incubated with MET (0, 0.1, 1, and 10 mM) for 2 h, followed by stimulation with MGO (200 μM) for 1 h. The protein concentrations of the cell lysates were measured using a BCA protein assay. Then, the levels of SOD, CAT, and GSH-Px were determined with the respective kits according to the manufacturer’s instructions.

### Determination of MMP (ΔΨm)

MMP was determined by JC-1 staining as described previously. HUVECs (1 × 10^5^ cells/well) were pretreated with MET (0, 0.1, 1, and 10 mM) for 2 h, followed by stimulation with MGO (200 μM) for 1 h. The cells were incubated with JC-1 (10 μg/mL) for 20 min at 37 °C in the dark and washed with PBS twice. Subsequently, the fluorescence intensity of JC-1 monomers and aggregates was measured at 490/530 and 525/590 nm, respectively, using a Spectra Max M5 microplate reader. The ratio of the fluorescence intensity of JC-1 monomers to aggregates was calculated to assess the change in MMP.

### Morphological observation of mitochondria

Morphological abnormalities in HUVEC mitochondria were observed under transmission electron microscopy (TEM). HUVECs were fixed with 2.5% glutaraldehyde, stained with cacodylate-buffered osmium tetroxide, and embedded in epoxy resin. Sections were prepared and examined using an electron microscope (Philips CM10, Philips, Eindhoven, Netherlands).

### Western blot analysis

The cells (1 × 10^5^ cells/well) were plated in 6-well plates under appropriate growth conditions, pretreated with MET (0, 0.1, 1, and 10 mM) for 2 h, and stimulated with MGO (200 μM) for 24 h. Cells were harvested and lysed by sonication in RIPA buffer supplemented with protease and phosphatase inhibitors (ThermoFisher Scientific, Shanghai, China) as previously described [27, 28]. The lysates were centrifuged at 16,000 rpm for 15 min at 4 °C, and then the supernatants were collected for further analysis. After the BCA assay, supernatants containing 25 μg of protein were subjected to 10% or 12% SDS/PAGE and transferred onto 0.22-μm PVDF membranes (BioRad, CA, USA). After blocking, the membranes were incubated with primary antibodies, rabbit or mouse IgG raised against Bax, Bcl-2, cleaved caspase-3, Nrf2, HO-1, phospho-Akt, Akt, and β-Actin, and the appropriate secondary antibodies (horseradish peroxidase-conjugated goat IgG raised against rabbit or mouse IgG) were used. Details of the above antibodies, including source and dilution, are provided in Table 1. Blots were developed using ECL substrate for detection (Pierce, Rockford, IL, USA). The band size and density analyses of western blots were performed using ImageJ software.

**Table 1:** Antibodies and dilutions used for western blotting.

Antibodies	MW (kDa)	Source and Cat. No.	Dilutions
*Primary*			
Phospho-Akt (Ser473)	60	Cell Signaling Technology, Beverly; #9271	1:1000
Akt	60	Cell Signaling Technology, Beverly; #4060	1:1000
Bcl-2	26	Cell Signaling Technology, Beverly; #15071	1:1000
Bax	20	Cell Signaling Technology, Beverly; #2772	1:1000
Cleaved Caspase-3 (Asp175)	19	Cell Signaling Technology, Beverly; #9661	1:500
Nrf2	60	Santa Cruz Biotechnology, CA; sc-365949	1:500
HO-1	32	Santa Cruz Biotechnology, CA; sc-136960	1:500
β-Actin	43	Beyotime Biotechnology, Shanghai; AF0003	1:1000
*Secondary*			
Horseradish peroxidase-conjugated goat anti-mouse IgG antibody raised anti-rabbit or mouse IgG		Beyotime Biotechnology, Shanghai; A0208/A0216	1:1000

*Nrf2* nuclear factor erythroid 2-related factor 2, *HO-1* heme oxygenase-1.

### Animals

C57/BL6 male mice, 12 weeks old, were purchased from SPF (Beijing) Biotechnology Co., Ltd. (Beijing, China). All mice were housed and acclimatized for 1 week before the experiments; the animals were maintained in a well-ventilated animal transit room with a 12 h light/dark cycle, relative humidity of 60 ± 10%, and a controlled temperature of 22 °C. The mice were fed standard laboratory rodent chow and given unrestricted access to food and tap water. Protocols for animal use were reviewed and approved by the Animal Care Committee of Southwest Medical University in accordance with Institutional Animal Care and Use Committee guidelines.

### MGO and MET administration

MGO was administered intraperitoneally to mice once daily for five consecutive days each week for 7 consecutive weeks. The initial dose administered was 50 mg/kg of bodyweight for the first 2 weeks, followed by a dose of 60 mg/kg for 3 weeks and a dose of 75 mg/kg for the last 2 weeks [29]. MET (300 mg/kg/day) was administered intragastrically every day for the last 5 weeks. MGO and MET were dissolved in 0.9% saline and normal drinking water, respectively, and placed at 4 °C in the dark. The animals were randomly divided into four groups: the vehicle group, MET group, MGO group, and MGO + MET group. The vehicle group was injected with 0.9% saline. Bodyweight, food intake, and blood glucose levels were monitored throughout the study period.

### Detection of MGO concentrations and inflammatory factor levels

All mice from each group were sacrificed by cervical dislocation, and the blood was collected. MGO concentrations in mouse serum samples were assayed with ELISA kits under the guidance of instructions. Pro- and anti-inflammatory cytokine (IL-1β, IL-6, and IL-10) levels in the serum were measured with R&D and ELISA kits according to the manufacturer’s instructions.

### Detection of MGO concentrations and inflammatory factor levels

MGO concentrations in mouse serum samples were assayed with ELISA kits under the guidance of instructions. Pro- and anti-inflammatory cytokine (IL-1β, IL-6, and IL-10) levels in the serum were measured with R&D and ELISA kits according to the manufacturer’s instructions.

### Immunohistochemical analysis and quantification

The aorta samples were fixed in 10% neutral-buffered formalin and processed to 5-μm thick sections for immunohistochemical staining. The slides were deparaffinized in xylene and subsequently rehydrated with graded alcohol, washed with ddH2O and finally in PBS, and incubated with 3% H_2_O_2_ for 15 min to quench endogenous peroxidase activity at room temperature. Subsequent antigen retrieval was completed by boiling sodium citrate buffer (PH 6.0) for 10 min. Then slides were incubated with normal goat serum for 15 min, followed by incubation with primary antibodies against cleaved caspase-3 at 1:200 dilution (#9661, cell signaling technology, USA), Nrf2 at 1:200 dilution (16396-1-AP, Proteintech Group, China), and phospho-Akt at 1:100 dilution (#4060, cell signaling technology, USA) at 4 °C overnight in a moist chamber. After being washed three times with PBS, the secondary antibody (Beyotime Biotechnology, China) was applied for 1 h at room temperature. Immunoreactivity was visualized by incubation with DAB (Beyotime Biotechnology, China). Hematoxylin was used for background counterstaining. For quantification, the semi-quantitative immunohistochemical score was obtained by examining at least five random fields per section under ×400 magnification and with digital image analysis by using ImageJ software.

### Statistical analysis

The data are expressed as the mean ± SD. Comparisons between two groups were made with an unpaired *t*-test, and comparisons between more than two groups were made with one-way ANOVA followed by the Bonferroni post-test. Statistical analyses were performed using GraphPad software (GraphPad Software, San Diego, CA, USA). A probability value of less than 0.05 was regarded as statistically significant.

## Results

### Effects of MET on the viability and apoptosis of MGO-injured HUVECs

We first examined the effect of MET on the viability of MGO-injured HUVECs by the MTT assay. As shown in Supplementary Fig. S1A, exposing HUVECs to MET at 20 and 50 mM for 2 h resulted in a significant reduction of cell viability, while no obvious side effects were observed after treatment with MET at 0.1, 1, and 10 mM. Furthermore, we found that treatment with MGO at 50, 100, 200, and 500 μM for 24 h significantly inhibited the viability of HUVECs (Supplementary Fig. S1B), which was in accordance with some previous studies [30]. When given in combination with MET, as shown in Supplementary Fig. S1C, pretreatment with 0.1, 1, and 10 mM MET for 2 h reversed MGO-mediated inhibition of cell viability in a dose-dependent manner in HUVECs.

**Fig. 1:**
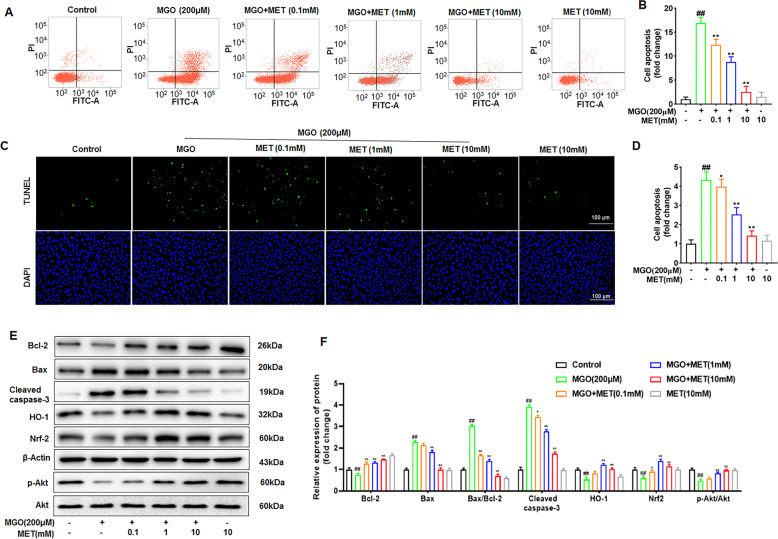
Suppression of MGO-induced apoptosis by MET. Cells were treated with different concentrations of MET (0, 0.1, 1, and 10 mM) for 2 h prior to being exposed to 200 μM MGO for 24 h. **A**, **B** Cells were trypsinized and stained with Annexin V-FITC and PI for 20 min. Apoptosis was analyzed by flow cytometry, and a representative image of the cell population distribution is displayed. **C**, **D** Cell apoptosis was examined by the TUNEL assay. Representative images of cell apoptosis are shown. Scale bars, 100 μm. The number of TUNEL-positive nuclei per field was counted in five randomly micrographs for each sample. Positive cells were counted, and quantitative assessment of quadruplicate cell apoptosis experiments was performed. **E**, **F** The effects of MET on MGO-induced changes of apoptosis-related proteins of Bax, Bcl-2, and cleaved caspase-3, as well as oxidation-related proteins of Nrf2 and HO-1 were examined by western blotting. β-Actin was used as a loading control. Moreover, the phosphorylated and total expression levels of Akt were also investigated by western blotting. Bax/Bcl-2 ratio quantified by densitometry and calculated with reference to β-Actin. All graphs correspond to the blots above them and represent densitometric analyses of three independent experiments; data are expressed as the mean ± SD. ^##^*p* < 0.05 vs. Control, *p < 0.05 vs^.^ MGO, **p < 0.01 vs. MGO.

To investigate the effect of MET on MGO-induced apoptosis, HUVECs were pretreated with various concentrations (0, 0.1, 1, and 10 mM) of MET for 2 h, followed by stimulation with MGO (200 μM) for 24 h. Then, flow cytometry with Annexin V-FITC and PI double staining was performed. Treatment of cells with MGO markedly increased the number of apoptotic cells relative to that in the untreated control group; moreover, the enhanced apoptosis was significantly suppressed by MET, particularly at 10 mM (Fig. 1A, B). Similarly, as shown in Fig. 1C, D, the TUNEL staining results also showed that MGO observably increased apoptosis, and this effect was evidently suppressed by MET treatment in a dose-dependent manner.

To determine the effect of MET on MGO-induced changes in antiapoptotic and proapoptotic proteins, Bax, Bcl-2, and cleaved caspase-3 were examined by western blotting. As shown in Fig. 1E, F, Bax and cleaved caspase-3 levels increased noticeably, whereas MGO decreased Bcl-2 levels compared to those in the control group. Treatment with MET decreased the expression of Bax and cleaved caspase-3 and increased the expression of Bcl-2 in a dose-dependent manner, thus attenuating the increase in the Bax/Bcl-2 ratio (Fig. 1E, F), with the maximal effect seen at a 10 mM concentration.

### MET prevents MGO-mediated impairment of the PI3K/Akt and Nrf2/HO-1 pathways

The PI3K/Akt pathway and Nrf2/HO-1 pathway play pivotal roles in the survival of certain cell types and are involved in the adaptive response to oxidative stress [31, 32]. Moreover, MGO-induced reduction of Akt phosphorylation has been considered to be closely related to cell apoptosis [33]. A previous study from our research group showed that Akt phosphorylation was decreased by MGO treatment as early as 30 min and then declined to a nadir after 60 min [34]. To understand the mechanisms of action of MET in MGO-induced apoptosis, the phosphorylated and total expression levels of Akt, Nrf2, and HO-1 were investigated by western blotting. The data showed that MGO treatment of HUVECs significantly reduced the phosphorylation of Akt and thus reduced the expression levels of Nrf2 and HO-1 (Fig. 1E, F). Furthermore, preincubation with MET at various concentrations clearly abrogated the MGO-mediated reductions in Akt phosphorylation and Nrf2/HO-1 protein levels (Fig. 1E, F). These data linked MGO-induced apoptosis and the PI3K/Akt/Nrf2/HO-1 signaling pathway, which was modulated by MET.

### MET suppresses MGO-induced intracellular ROS generation

**Fig. 2:**
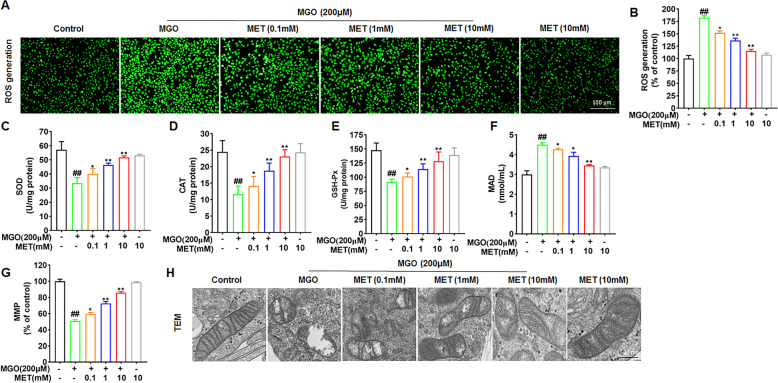
MET decreases MGO-induced intracellular ROS generation. HUVECs were treated with MET (0, 0.1, 1, and 10 mM) for 2 h and then exposed to MGO (200 μM) for 1 h. **A**, **B** Then, the cells were stained with DCFH-DA, and the fluorescence intensity was measured at 488/525 nm using a microplate reader. Scale bar, 100 μm. **C**–**F** The levels of SOD, CAT, and GSH-Px and the MDA content were measured with the respective ELISA kits. **G** The MMP was assessed with the JC-1 probe. The fluorescence intensities of JC-1 monomers (490/530 nm) and JC-1 aggregates (525/590 nm) were measured using a microplate reader. The ratio of JC-1 aggregates/JC-1 monomers was calculated. **H** Ultrastructural alterations of mitochondria were detected by TEM. Scale bars: 500 nm. All data are shown as the mean ± SD of three independent experiments. ^##^*p* < 0.01 vs. Control, *p < 0.05 vs. MGO, **p < 0.01 vs. MGO.

ROS have been shown to be involved in regulating cell apoptosis and are strictly controlled by an antioxidant program [35, 36]. To determine the effects of MET on MGO-induced intracellular ROS generation, HUVECs were treated with MET (0, 0.1, 1, and 10 mM) for 2 h and then exposed to MGO (200 μM) for 1 h. As shown in Fig. 2A, B, MGO significantly increased ROS production in HUVECs. Treatment with MET suppressed ROS production in HUVEC in a dose-dependent manner, especially in the presence of 10 mM MET, which reduced the production of ROS to levels comparable to those of non-MGO-treated control. Furthermore, the activity of antioxidant enzymes, including SOD, CAT, and GSH-Px, was measured. The data showed that MGO induced a clear decrease in SOD, CAT, and GSH-Px activities; however, treatment with MET remarkably increased the downregulated antioxidant enzyme activities induced by MGO in a dose-dependent manner, particularly at 10 mM (Fig. 2C–E). As shown in Fig. 2F, the MDA content was also measured. Treatment of cells with MGO significantly increased the MDA content but this content was restored with MET pretreatment. These results suggest that MET prevents MGO-induced oxidative stress.

### MET prevents MGO-induced mitochondrial damage

The induction of apoptosis is thought to be mediated by mitochondrial dysfunction, which is reflected by the MMP [37]. Previous studies have shown that MGO induces the opening of permeability transition pores (PTPs) and thus evidently reduces MMP levels [38, 39]. Here, as shown in Fig. 2G, our data show that stimulation of cells with MGO markedly reduced MMP levels compared to those in the control group, suggesting that the MMP was depolarized. Preincubation with MET inhibited MGO-induced MMP depolarization in a dose-dependent manner.

Furthermore, mitochondrial morphology was analyzed and imaged by using TEM. As shown in Fig. 2H, HUVECs in the control group showed normal mitochondria with preserved membranes and cristae. After treatment of cells with MGO (200 μM) for 1 h, damaged mitochondria were observed, with the outer membranes and cristae showing a loss of ultrastructural integrity. Notably, treatment with MET protected against mitochondrial morphological alterations in a dose-dependent manner, suggesting that MET can prevent MGO-induced mitochondrial damage.

### Effects of NAC and CsA on MGO-induced HUVEC apoptosis and ROS generation

**Fig. 3:**
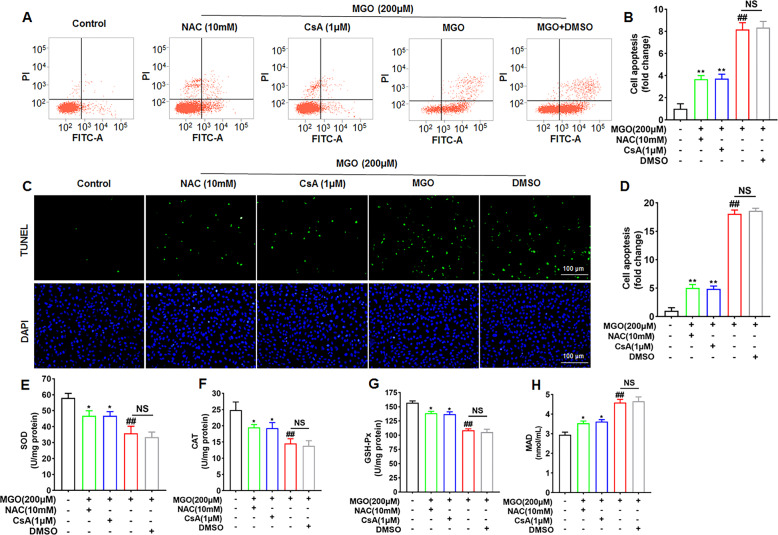
Effects of NAC and CsA on MGO-induced HUVEC apoptosis. HUVECs were pretreated with NAC (10 mM) and CsA (1 μM) for 2 h, followed by stimulation with MGO for 24 h. **A**, **B** Cell apoptosis was analyzed by flow cytometry based on Annexin V-FITC and PI double staining. **C**, **D** Cell apoptosis was examined by TUNEL assays. Scale bars, 100 μm. Representative images of cell apoptosis are shown. **E**–**H** The levels of SOD, CAT, GSH-Px, and MAD were measured with the respective kits according to the manufacturer’s instructions. The values (mean ± SD from three independent experiments) are relative to the control and are expressed as fold changes. ^##^*p* < 0.01 vs. Control, *p < 0.05 vs. MGO, **p < 0.01 vs. MGO.

To confirm whether inhibition of mitochondrial dysfunction is associated with antiapoptotic effects, HUVECs were pretreated with CsA (1 μM, an inhibitor of the mammalian PTP) for 2 h, followed by stimulation with MGO for 24 h, and cell apoptosis was measured. As shown in Fig. 3A, B, CsA significantly inhibited MGO-induced cell apoptosis, indicating that the protective effects of MET on cell apoptosis are involved in the inhibition of mitochondrial dysfunction.

Furthermore, to investigate the effects of antioxidants on MGO-induced cell apoptosis and ROS generation, HUVECs were pretreated with NAC (10 mM) for 2 h and then stimulated with MGO for 24 h. NAC, a ROS scavenger, was used. As shown in Supplementary Fig. S2A, B, MGO-induced ROS production in HUVEC was remarkably decreased in NAC group. Moreover, flow cytometry analysis showed that NAC significantly attenuated MGO-induced apoptosis of HUVECs (Fig. 3A, B). The TUNEL assay also showed that NAC reversed the cell apoptosis induced by MGO (Fig. 3C, D). In addition, NAC had a statistically significant inhibitory effect on the increases in MDA content and antioxidant enzyme (SOD, CAT, and GSH-Px) activities induced by MGO (Fig. 3E–H). These results indicated that NAC significantly inhibited MGO-induced HUVEC apoptosis and ROS generation.

### MET suppresses MGO-induced apoptosis and Akt dephosphorylation

**Fig. 4:**
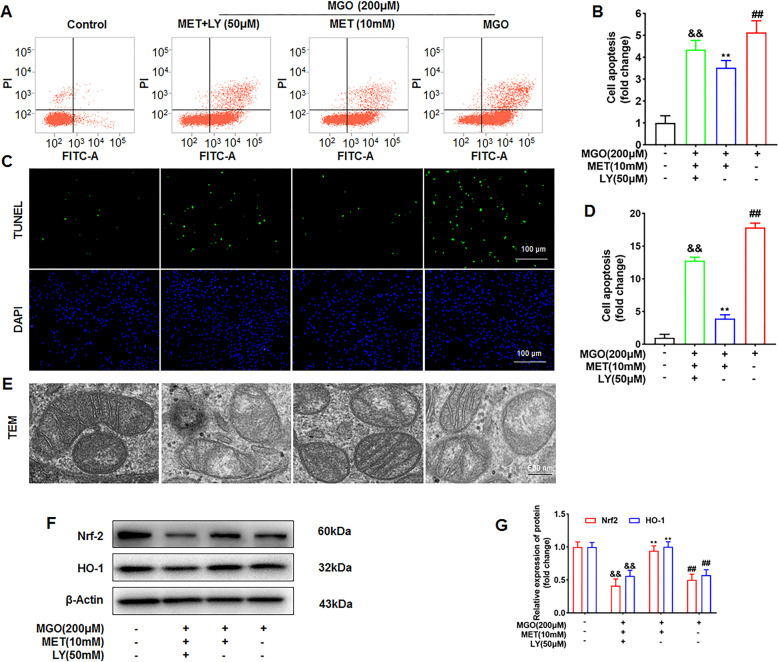
MET suppresses MGO-induced apoptosis and Akt dephosphorylation. HUVECs were pretreated with MET (10 mM), LY-294002 (LY, 50 μM, a PI3K inhibitor), and the vehicle control for 2 h, followed by stimulation with MGO for 24 h. **A**, **B** Cell apoptosis was analyzed by flow cytometry based on Annexin V-FITC and PI double staining. **C**, **D** Cell apoptosis was also examined by the TUNEL assay. Scale bars, 100 μm. Representative images of cell apoptosis are shown. **E** Ultrastructural alterations of mitochondria were detected by TEM. Scale bars: 500 nm. **F**, **G** Representative western blots of total cell lysates after immunoblotting and probing with antibodies against Nrf2 and HO-1, which were quantified by densitometry and calculated as ratios to β-Actin. The values (mean ± SD from three independent experiments) are relative to the control and are expressed as fold changes. ^##^*p* < 0.01 vs. Control, **p < 0.01 vs. MGO, ^&&^*p* < 0.01 vs. MGO + MET.

To determine the importance of Akt dephosphorylation in MGO-induced apoptosis and investigate whether this effect was suppressed by MET, we pretreated HUVECs with MET (10 mM), LY (50 μM, the PI3K inhibitor), and vehicle control for 2 h, followed by stimulation with MGO for 24 h. As shown in Fig. 4A, B, LY significantly attenuated the protective effects of MET on MGO-induced apoptosis. TUNEL staining also generated similar results (Fig. 4C, D). Next, we investigated the effect of LY on mitochondrial morphological alterations. Figure 4E revealed that LY counteracted the protective effect of MET on mitochondria morphology. In addition, the levels of Nrf2 and HO-1 were evaluated with the inhibitor LY by western blotting. As shown in Fig. 4F, G, pretreatment with MET increased the levels of Nrf2 and HO-1 compared to MGO treatment. Conversely, treatment with LY antagonized some of the antioxidant effects of MET and decreased Nrf2 and HO-1 expression based on the combined use of MGO and MET. As expected, those data confirmed that stimulation with LY markedly attenuated the protective effects of MET on Nrf2/HO-1 signaling.

Furthermore, to investigate whether Nrf2 signaling was involved in the activity of MET, we pretreated HUVECs with an Nrf2 inhibitor (ML385, 20 mM) for 2 h, followed by stimulation with MGO for 24 h. As Supplementary Fig. S3A shows, the decreased viability of HUVECs induced by MGO was evidently reversed by MET; however, ML385 markedly attenuated the protective effect of MET on the viability of HUVECs. Moreover, ML385 significantly suppressed the effect of MET on MGO-induced inhibition of the Nrf2/HO-1 pathways (Supplementary Fig. S3B, C). Similar results were also confirmed by TUNEL staining, as shown in Supplementary Fig. S3D, E. ML385 notably inhibited the protective effect of MET on MGO-induced HUVEC apoptosis. The above results indicate that the protective effects of MET against MGO-induced cell apoptosis are partly mediated through the PI3K/Akt and Nrf2/HO-1 signaling pathways.

### Effects of MET and MGO administration on physiological changes, oxidative indexes, and inflammatory factors in mice

**Fig. 5:**
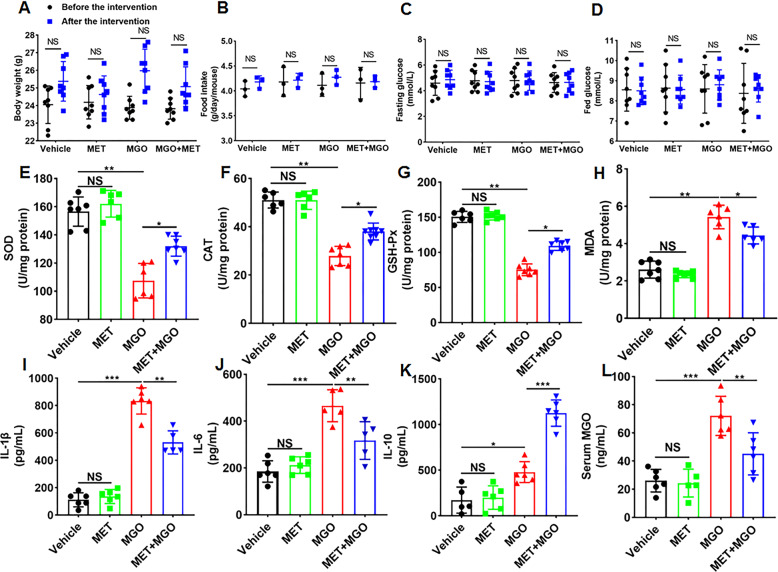
Effects of MET on MGO-treated mice. C57BL/6 mice were treated with MGO and MET, and physiological and biochemical characteristics were collected regularly and analyzed. At the end of the experiment, blood samples from the mice were collected for analysis. **A** Bodyweight. **B** Food intake. **C** Fasting blood glucose. **D** Fed glucose. **E**–**G** The levels of SOD, CAT, and GSH-Px were measured with the respective kits according to the manufacturer’s instructions. **H** The MDA content was also measured. **I**–**K** Changes in the pro-inflammatory cytokines IL-1β and IL-6 and the anti-inflammatory cytokine IL-10 were determined. **L** The MGO ELISA Kit was used to detect the MGO level in the serum. Data are presented as the mean ± SD, *n* ≥ 5 for each group. *p < 0.05, **p < 0.01, ***p < 0.001, NS: not significant.

To determine whether MET affects MGO-induced vascular injury in vivo, C57BL/6 mice were treated with MGO and MET. At the end of the experiment, blood samples and tissues from the mice were collected for analysis. Interestingly, there were no significant changes in bodyweight, food intake, fasting blood glucose or fed glucose before and after treatment in the drug-treated mice compared with the vehicle-treated mice (Fig. 5A–D). As shown in Fig. 5E–H, MGO stimulated significant decreases in the levels of SOD, CAT, and GSH-Px while causing increases in MDA, but these levels were only partially restored with MET pretreatment. These results are in good agreement with those obtained from the in vitro studies, as shown in Fig. 2C–F, suggesting that MET suppressed MGO-induced oxidative stress in vivo and in vitro.

Furthermore, dynamic changes in the levels of the pro-inflammatory cytokines IL-1β and IL-6 and the anti-inflammatory cytokine IL-10 were investigated. As shown in Fig. 5I–K, MGO observably aggrandized IL-1β and IL-6 levels, while IL-10 levels were abated. Treatment with MET remarkably increased the downregulated IL-10 levels but significantly decreased the upregulated IL-1β and IL-6 levels. To detect the absorption of MGO in mice, the concentration of MGO in mouse serum was determined. MGO serum levels were increased by ∼3-fold compared with those of vehicle-treated mice (Fig. 5L). These data indicate the in vivo anti-inflammatory activity of MET against MGO-induced inflammation.

### MET prevents MGO-induced apoptosis by regulating the PI3K/Akt and Nrf2/HO-1 pathways in vivo

**Fig. 6:**
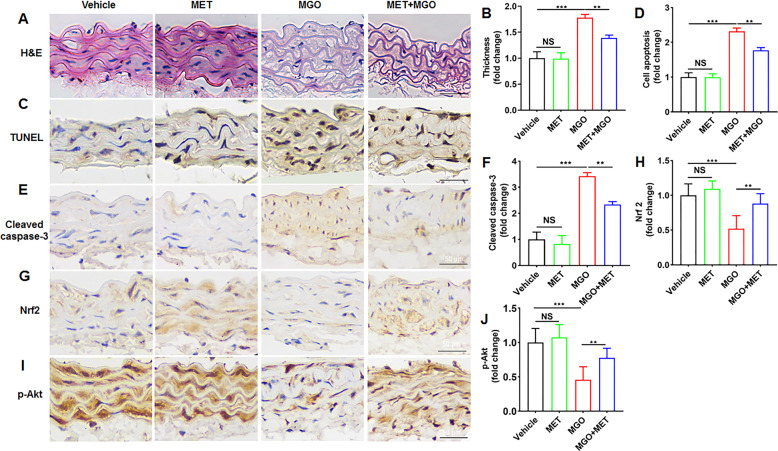
MET prevents MGO-induced apoptosis by modulating the PI3K/Akt and Nrf2/HO-1 pathways in vivo. C57BL/6 mice were treated with MGO and MET, after which the aortas were collected for biochemical parameters. **A**, **B** Histological changes in the aortas were evaluated by H&E staining. **C**, **D** Apoptosis was evaluated by TUNEL staining. **E**, **F** The proapoptotic factor cleaved caspase-3 was evaluated by Immunohistochemistry staining. **G**, **H** Nrf2 expression was evaluated by Immunohistochemistry staining. **I**, **J** The phosphorylation of Akt was also determined by Immunohistochemistry staining. Data are presented as the mean ± SD, *n* ≥ 5 for each group. **p < 0.01, ***p < 0.001. Bar = 50 μm.

The in vivo effect of MET on MGO-induced apoptosis was determined via oral administration in C57BL/6 mice. Aortas were examined pathologically with H&E staining (Fig. 6A, B), which showed an increase in aortic thickening after stimulation with MGO for 7 weeks. Treatment with MET prevented MGO-induced pathological alterations. The TUNEL assay demonstrated that MET reversed the apoptosis induced by MGO in vivo (Fig. 6C, D). To further examine the preventive effect of MET on MGO-induced apoptosis, immunohistochemical staining showed the increased expression of a proapoptotic factor, cleaved caspase-3. Treatment with MET evidently prevented the increase in the levels of cleaved caspase-3 induced by MGO in the aortas (Fig. 6E, F). To elucidate the underlying mechanisms, we measured Nrf2 expression and Akt phosphorylation (p-Akt) with immunohistochemistry staining. As shown in Fig. 6G–J, Nrf2 expression and phosphorylation of Akt were notably decreased in the aorta of MGO-induced mice but significantly increased by MET treatment. As expected, MET reversed the MGO-induced downregulation of Nrf2 and p-Akt.

## Discussion

MGO is a highly reactive dicarbonyl metabolite of glucose known to induce cellular apoptosis, oxidative stress, inflammation, and AGE formation in ECs [40, 41]. The accumulation of MGO has been associated with organ dysfunction contributing to the pathogenesis of diabetes and vascular complications [42, 43]. MET appears to reduce excessive ROS generation and cell apoptosis [44]. However, the specific involvement of MET in MGO-induced apoptosis in HUVECs and the underlying signaling pathways have remained uncertain. Our results presented here confirm that MET can prevent MGO-induced HUVEC apoptosis in vitro and in vivo by decreasing oxidative stress, mitochondrial damage, and inflammatory reactions and increasing antioxidant levels, which is associated with activation of the PI3K/Akt and Nrf2/HO-1 signaling pathways.

MGO has been reported to decrease cell viability and induce cellular apoptosis in multiple cell types [45, 46], including HUVECs [47]. Our present data clearly demonstrated that MGO treatment significantly reduced HUVEC viability and increased HUVEC apoptosis and that MET protected HUVECs from MGO-induced apoptosis in a dose-dependent manner. Consistent with these observations, pretreatment with MET prevented MGO-induced HUVEC apoptosis, inhibited the elevation of the Bax/Bcl-2 ratio, and attenuated the activation of cleaved caspase-3 in a dose-dependent manner, thus confirming the cytoprotective effects of MET against MGO-induced apoptosis. The in vivo effect of MET on MGO-induced apoptosis was determined by using an MGO-induced vascular injury model in mice, and our data also demonstrated that MET prevented apoptosis induced by MGO by inhibiting the activation of cleaved caspase-3. These findings were consistent with previous studies [33, 48], indicating that MET effectively prevented HUVEC apoptosis, providing novel insights into the mechanisms of action of MET.

MGO has been shown to induce ROS generation and has a significant adverse effect on the antioxidant defense system [49]. Data obtained in the present study show that pretreatment with MET markedly inhibited MGO-induced ROS generation and caused a clear decrease in SOD, CAT, and GSH-Px activities in HUVECs. Our in vivo data also indicated that the levels of SOD, CAT, and GSH-Px were decreased by stimulation with MGO but were only partially restored by pretreatment with MET. Previous studies have shown that ROS production is associated with apoptosis induction [50]. Here, we found that preincubation with the antioxidant NAC notably inhibited ROS generation and MGO-induced apoptosis. From these data, we concluded that MET inhibits MGO-induced apoptotic biochemical changes by blocking ROS formation in vivo and in vitro.

The generation of ROS, disruption of MMP, and mitochondrial dysfunction are characteristic features of intracellular apoptosis [51]. The present study showed that MET inhibited MGO-induced depolarization of MMP and protected against mitochondrial morphological alterations in a dose-dependent manner. In addition, it was previously reported that the initiation of apoptosis is regulated by Bcl-2 family proteins through the maintenance of mitochondrial PTP opening [52]. Our results are in good agreement with previous studies showing that PTP opening is regulated by both the elevation of the Bax/Bcl-2 ratio and caspase-3 activation, consequently inducing cell apoptosis, which indicates that a protective mechanism of MET in the context of mitochondrial function is associated with inhibition of mitochondrial PTP opening. In addition, by using an inhibitor of PTP opening (CsA), our experimental results indicated that loss of MMP inhibited MGO-induced apoptosis. Similar results had been reported previously [34]. MDA, a product of lipid peroxidation by ROS, is commonly used as a biomarker of oxidative stress. Here, we present both in vitro and in vivo evidence that MET exerts its protective effects against cell damage by inhibiting the release of MDA. Collectively, these findings strongly indicate that ROS generation triggered by MGO is blocked by MET and support the hypothesis that MET prevents MGO-induced apoptosis through protective effects on mitochondrial function.

Previous studies have shown that Nrf2 is a transcription factor that upregulates antioxidant genes such as HO-1 and plays a key role in defense against oxidative stress [53]. HO-1, an antioxidant enzyme, is induced in response to oxidative stress and is regulated by the Nrf2 pathway [54]. In addition, Akt, which is downstream of PI3K, is considered to exert antioxidant and antiapoptotic effects by enhancing the transcriptional activity of Nrf2 and then inhibiting its interaction with antiapoptotic Bcl-2 family members [33, 55]. Several studies have reported that the PI3K/Akt pathway is essential for regulating Nrf2/Ho-1 pathway activation and is thus involved in protection against oxidative stress and apoptosis in multiple cell types [56, 57]. Furthermore, in our previous studies, we showed that inhibition of Akt phosphorylation is associated with the regulation of MGO-induced cell apoptosis [34]. In the present study, pretreatment with MET attenuated the decrease in Akt phosphorylation in a dose-dependent manner, thus increasing Nrf2/HO-1 levels and suppressing MGO-induced apoptosis both in vitro and in vivo. Furthermore, the PI3K/Akt pathway inhibitor LY-294002 markedly abolished the antiapoptotic effect of MET and thus inhibited Nrf2/HO-1 signaling. Moreover, our results also demonstrated that ML385, an Nrf2 inhibitor, notably abolished the protective effect of MET on MGO-induced HUVEC apoptosis. Taken together, our findings clearly show that MET might exert its protective effects by upregulating the PI3K/Akt/Nrf2/HO-1 pathways.

MGO-induced ROS generation, cellular apoptosis, and inflammation are specific events in HUVECs that induce endothelial dysfunction [58]. Our in vivo results are further supported by previous studies [59–61], which demonstrated that MGO significantly increases levels of the pro-inflammatory cytokines IL-1β and IL-6, while levels of the anti-inflammatory cytokine IL-10 are decreased. Furthermore, our present study indicates that MET exerts anti-inflammatory effects against inflammation induced by MGO.

**Fig. 7:**
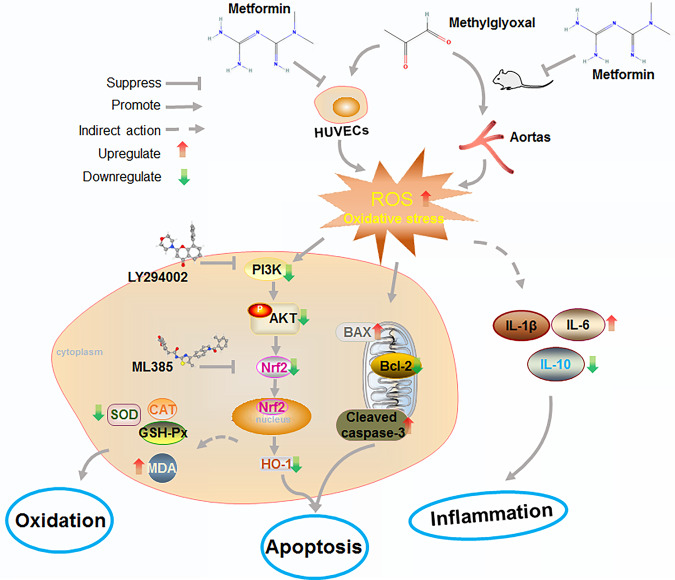
Schematic diagram showing the cytoprotective signaling associated with MET in MGO-induced endothelial cell apoptosis. The possible molecular mechanisms of MET in MGO-induced endothelial dysfunction in human ECs were explored. As depicted, MET inhibited the apoptotic signaling cascades initiated by MGO-generated ROS via modulating the PI3K/Akt and Nrf2/HO-1 signaling pathways. Furthermore, MET effectively exerts protective effects against MGO-induced oxidative stress, mitochondrial dysfunction, apoptosis, and inflammation in vitro and in vivo.

In conclusion, our study is the first to demonstrate that MET effectively exerts protective effects against MGO-induced oxidative stress, mitochondrial dysfunction, apoptosis, and inflammation in vitro and in vivo. Specifically, MET prevents the apoptotic signaling cascades initiated by MGO-generated ROS by modulating the PI3K/Akt and Nrf2/HO-1 signaling pathways (Fig. 7). This compelling evidence expands our understanding of the benefits and clinical applications of MET therapy, providing novel insights for the development of strategies to preserve endothelial function in diabetic vascular diseases.

## Supplementary information

Supplementary legends

FIG S1

FIG S2

FIG S3

The Reproducibility checklist

## Data Availability

All data generated or analyzed during this study are included in this published article and its additional files.
