# Supplementary material for: Metformin prevents methylglyoxal-induced apoptosis by suppressing oxidative stress in vitro and in vivo
Source: Cell Death Dis. 2022 Jan 10;13(1):29. doi: 10.1038/s41419-021-04478-x (PMC8748764; doi:10.1038/s41419-021-04478-x)
Supplement: Supplementary file 1 — Supplementary legends [file 41419_2021_4478_MOESM1_ESM.docx]

**Supplementary legends**

**Fig. S1 Effects of MET on the viability of MGO-injured HUVECs**. (A) HUVECs were treated with MET (0, 0.1, 1, 10, 20, and 50 mM) for 2 h. (B) HUVECs were treated with MGO at 10 μM, 50 μM, 100 μM, 200 μM and 500 μM for 24 h. (C) HUVECs were pretreated with (0, 0.1, 1, and 10 mM) MET for 2 h, followed by coincubation with 200 μM MGO for 24 h. Cell viability was determined by the MTT method. Values are presented as the mean ± SD from three independent experiments. ^#^p <0.05 vs. Control, ^##^p <0.01 vs. Control, *p <0.05 vs. MGO, ** p <0.01 vs. MGO.

**Fig. S2 NAC and CsA act on MGO-induced ROS generation.** (A, B) HUVECs were pretreated with NAC (10 mM), CsA (1 μM) and the vehicle control for 2 h, followed by stimulation with MGO (200 μM) for 1 h. The level of ROS generated was then measured by using the redox-sensitive dye DCF-DA, and the fluorescence intensity was measured at 488/525 nm using a microplate reader, recording typical pictures. The values (mean ± SD from three independent experiments) are presented as % of control (first bar). Scale bar, 100 μm. ^##^p <0.01 vs. Control, *p <0.05 vs. MGO, **p <0.01 vs. MGO.

**Fig. S3** **ML385 regulates the Intervention effects of MET.** HUVECs were pretreated with a Nrf2 inhibitor (ML385, 20 mM), MET (10 mM), and the vehicle control for 2 h, followed by stimulation with MGO for 24 h. (A) Cell viability was determined by the MTT method. (B and C) Representative Western blots of total cell lysates after immunoblotting and probing with antibodies against Nrf2 and HO-1, which were quantified by densitometry and calculated as ratios to β-Actin. (D and E) Cell apoptosis was examined by the TUNEL assay. Scale bars, 100 μm. Representative images of cell apoptosis are shown. The values (mean ± SD from three independent experiments) are relative to the control and are expressed as fold changes. ^##^p <0.01 vs. Control, **p <0.01 vs. MGO, ^&&^p <0.01 vs. MGO + MET.
