# Supplementary figures and images for: Metformin prevents methylglyoxal-induced apoptosis by suppressing oxidative stress in vitro and in vivo
Source: Cell Death Dis. 2022 Jan 10;13(1):29. doi: 10.1038/s41419-021-04478-x (PMC8748764; doi:10.1038/s41419-021-04478-x)

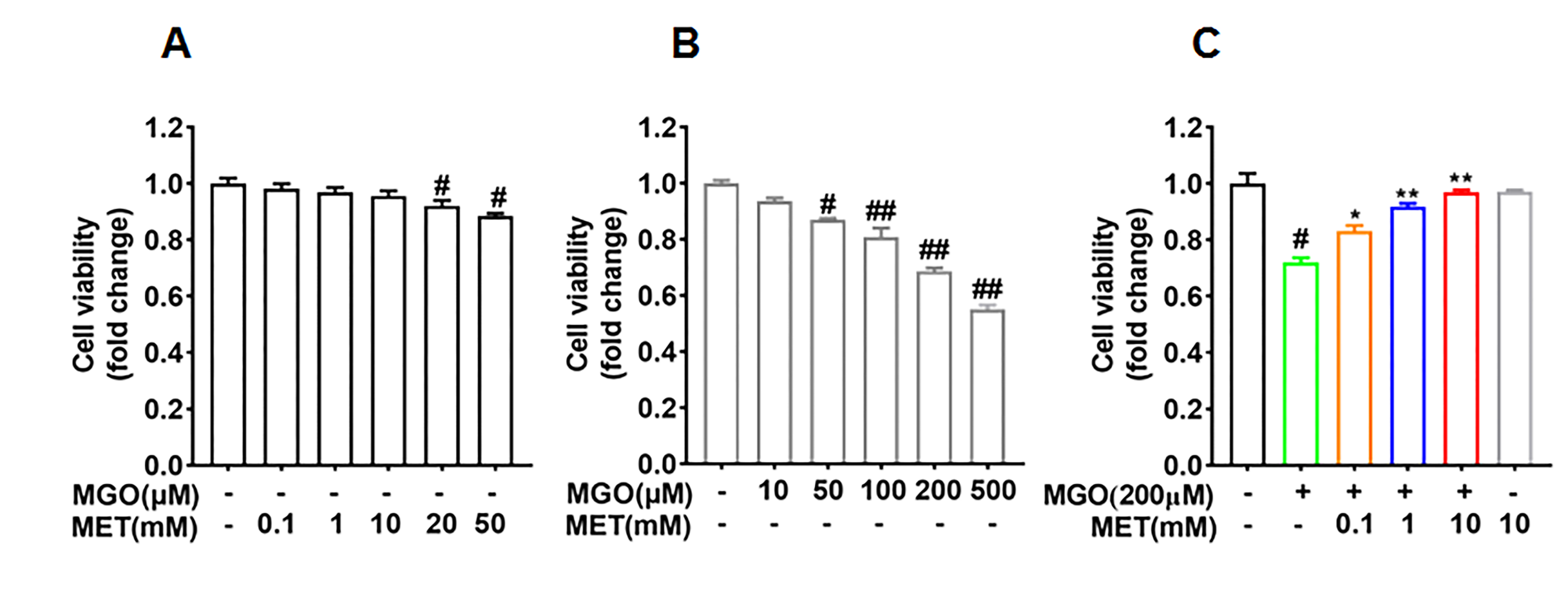

Supplement: Supplementary file 2 — FIG S1 [file 41419_2021_4478_MOESM2_ESM.tif]

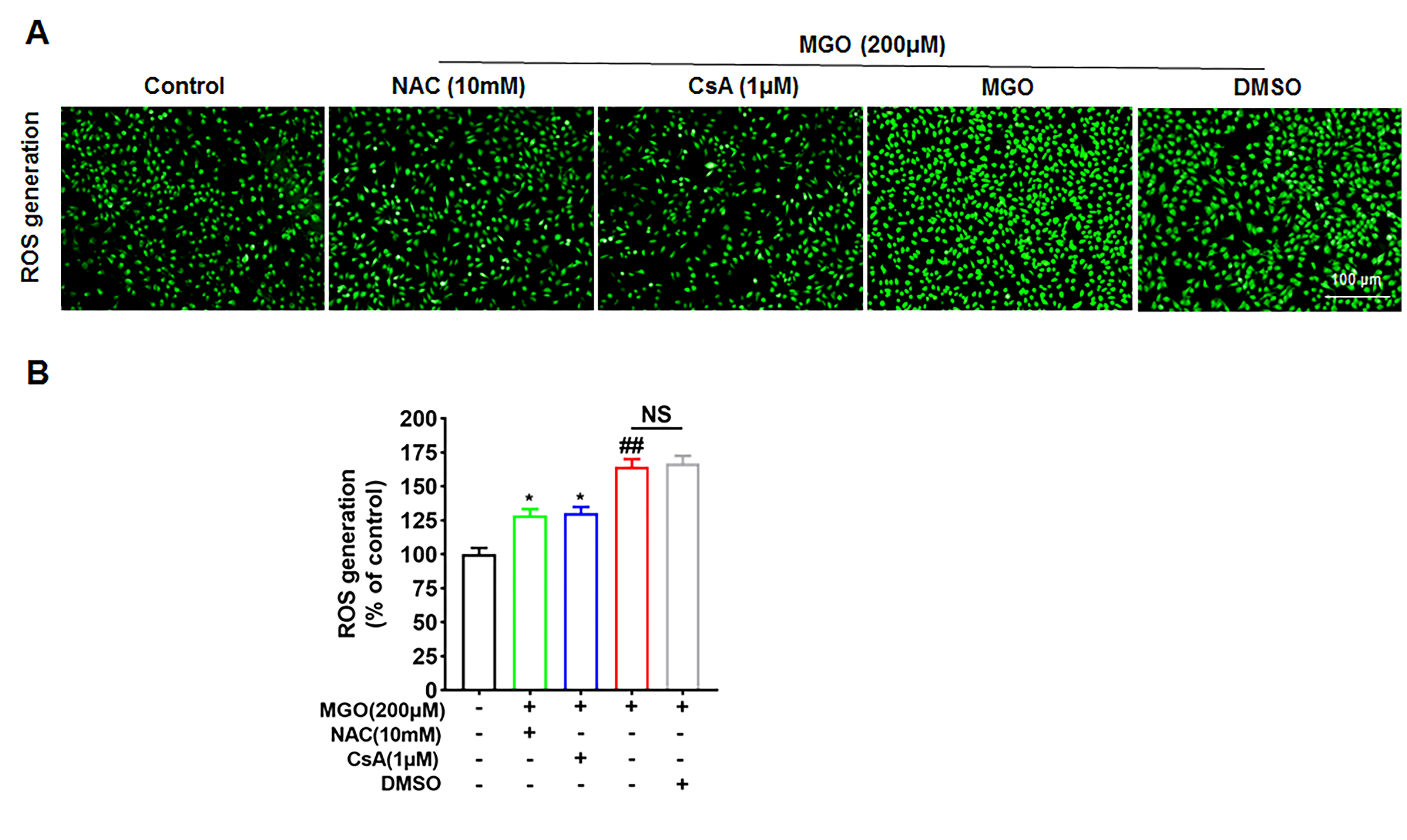

Supplement: Supplementary file 3 — FIG S2 [file 41419_2021_4478_MOESM3_ESM.tif]

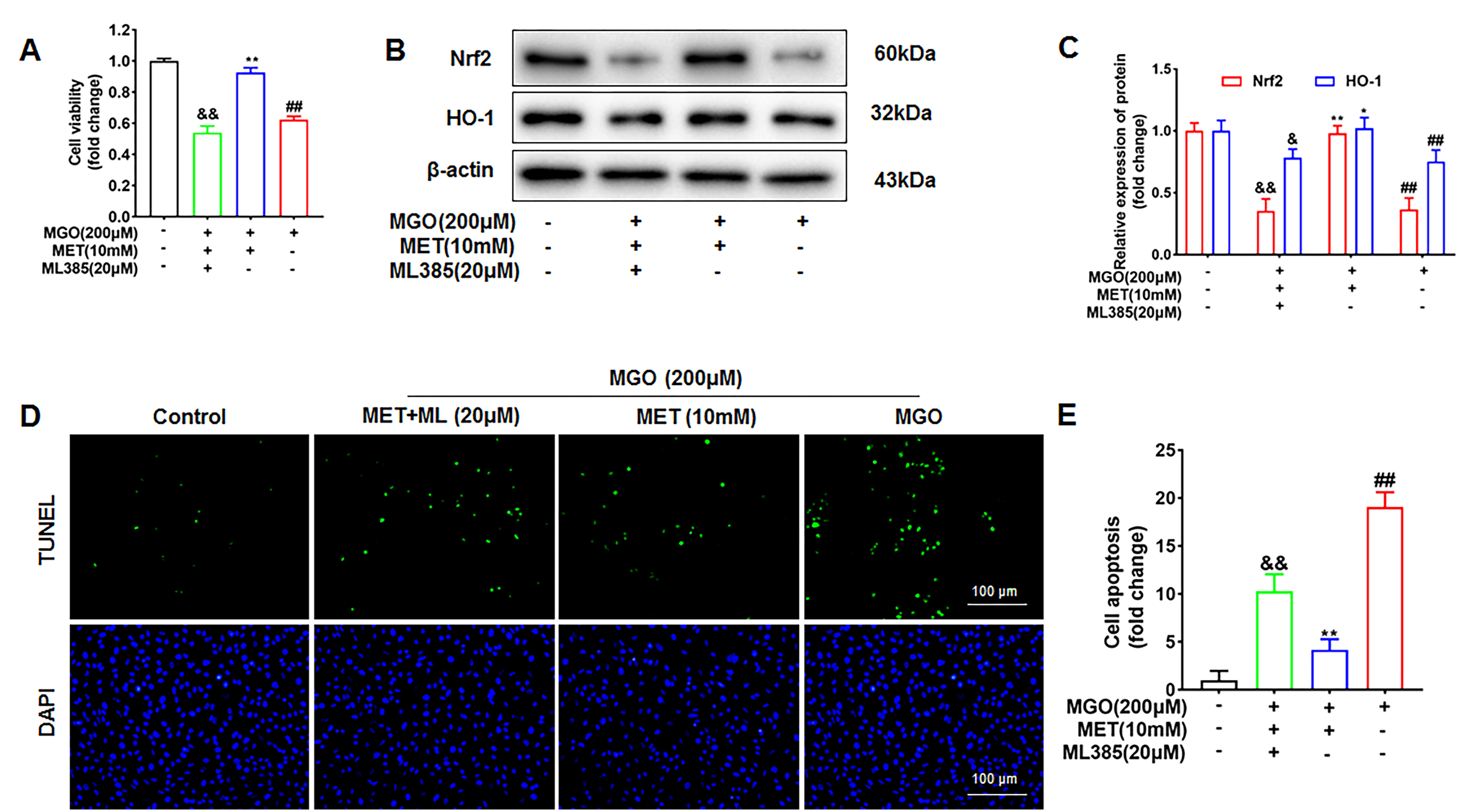

Supplement: Supplementary file 4 — FIG S3 [file 41419_2021_4478_MOESM4_ESM.tif]
